# Association of cancer progression with elevated expression of programmed cell death protein 1 ligand 1 by upper tract urothelial carcinoma and increased tumor-infiltrating lymphocyte density

**DOI:** 10.1007/s00262-020-02499-7

**Published:** 2020-02-06

**Authors:** Akinori Nukui, Takao Kamai, Kyoko Arai, Toshiki Kijima, Minoru Kobayashi, Takahiro Narimatsu, Tsunehito Kambara, Hideo Yuki, Hironori Betsunoh, Hideyuki Abe, Yoshitatsu Fukabori, Masahiro Yashi, Ken-Ichiro Yoshida

**Affiliations:** 1 Department of Urology, Dokkyo Medical University, 880 Kitakobayashi Mibu, Utsunomiya, Tochigi 321-0293 Japan; 2 Department of Urology, Utsunomiya Memorial Hospital, Utsunomiya, Tochigi Japan

**Keywords:** Programmed cell death 1 ligand 1 (PD-L1), Tumor-infiltrating lymphocyte (TIL), Upper tract urothelial carcinoma (UTUC), Neutrophil-to-lymphocyte ratio (NLR)

## Abstract

**Background:**

Increased expression of programmed cell death 1 ligand 1 (PD-L1) by tumor cells is thought to be a mechanism through which solid cancers promote immune tolerance. However, the association between PD-L1 expression and the prognosis of upper urinary tract urothelial carcinoma (UTUC) remains unknown.

**Methods:**

We examined immunohistochemical PD-L1 expression and the tumor-infiltrating lymphocyte density (TILD) in 79 patients with UTUC who underwent nephroureterectomy. We classified the tumors into four types based on the combination of PD-L1 expression and TILD, and studied the clinicopathological characteristics of these four tumor types.

**Results:**

Elevated expression of PD-L1 by tumor cells and a higher TILD were associated with a worse histological grade, higher pT stage, and higher peripheral blood neutrophil-to-lymphocyte ratio. Elevated expression of PD-L1 by tumor cells, a higher TILD, and type I, III, or IV tumors with elevated expression of either PD-L1 or TILD showed a positive correlation with poorer differentiation and local invasion. These three variables were associated with shorter progression-free survival and overall survival in univariate analysis, but only the latter was an independent determinant according to multivariate analysis. The patients who had type II tumors with lower PD-L1 expression and a lower TILD showed more favorable survival than the other three groups.

**Conclusions:**

These findings suggest that PD-L1 expression and TILs in the tumor microenvironment influence the progression of UTUC. Accordingly, it is important to understand the immunologic characteristics of the tumor microenvironment to develop more effective treatment strategies for this cancer.

**Electronic supplementary material:**

The online version of this article (10.1007/s00262-020-02499-7) contains supplementary material, which is available to authorized users.

## Introduction

Upper urinary tract urothelial carcinoma (UTUC) is a relatively uncommon tumor, accounting for < 10% of all urothelial malignancies, although its incidence has been increasing [1]. Many patients develop intravesical recurrence, lymph-node metastasis, and systemic metastasis within a few years after curative surgical resection, even following complete resection, presumably due to the presence of occult micrometastasis at the time of surgery and the thin walls and rich lymphatic drainage of the ureter [2, 3]. While intravesical recurrence can be controlled by transurethral resection, lymph-node or distant metastasis tends to be refractory to chemotherapy, eventually leading to unfavorable outcomes [4]. Metastatic urothelial cancer (UC) progresses rapidly, and the survival time is often less than 1 year after distant metastasis occurs [5]. Standard first-line chemotherapy for metastatic UC is the combination of gemcitabine and cisplatin (GC) or the combination of methotrexate, vinblastine, doxorubicin, and cisplatin (MVAC) [6, 7]. Both regimens initially achieve favorable response rates, but neither has much impact on the eventual prognosis [8–10]. Thus, there is an unmet need for more effective treatment.

Tumor cells must evade destruction by the immune system to survive and such behavior is currently considered to be one of the “hallmarks of cancer” [11]. The immune system is capable of suppressing tumor development and causing tumor regression, but can also stimulate tumor growth. These dual host-protective and tumor-promoting actions of the immune system are referred to as cancer immunoediting [12]. Although T cells have an important role in the host immune response to malignancy, T-cell-based anticancer immunotherapy is associated with several limitations, including the need to maintain self-tolerance and the requirement for inhibitory pathways to regulate the duration and amplitude of the immune response. It has been clarified that tumors utilize certain immune checkpoint pathways as a major mechanism of resistance to the host immune system. Generation and activation of tumor antigen-specific T cells are essential for the host immune system to display antitumor activity. Multiple co-stimulatory receptors and negative regulators (or co-inhibitory receptors) act in concert to control T-cell activation and proliferation, as well as regulating gain or loss of effector function [13–15]. One of the most promising approaches for promoting antitumor immunity is immune checkpoint blockade, because tumors utilize these checkpoints as a mechanism of resistance, particularly against T cells targeting tumor antigens [13]. Accordingly, blocking one or more of the immune checkpoints may be a promising approach for activation of antitumor immunity. The B7 and CD28 families play a pivotal role in the immune checkpoint system by activating and inhibiting co-stimulatory molecules that positively or negatively regulate immune responses [13–15]. Among the various molecules in these families, those in the programmed cell death protein 1 (PD-1)/PD-1 ligand 1 (PD-L1) pathway negatively regulate T-cell activation are important for controlling host antitumor immunity. One of the central methods through which tumors resist elimination by endogenous tumor-specific T cells is upregulation of PD-L1 expression, since PD-L1 suppresses T-cell migration/proliferation and also restricts cancer cell killing by prevention of binding to T-cell receptors [12, 13]. A strong association between higher levels of PD-L1 expression and adverse clinical outcomes has been demonstrated for various cancers, including UC [15]. Thus, the PD-1/PD-L1 pathway seems to be an attractive target for development of anticancer immunotherapy. PD-L1 is a transmembrane protein that downregulates antitumor responses by promoting apoptosis of tumor-infiltrating lymphocytes (TILs) and thus facilitates tumor progression [13–15]. Several recent clinical trials that targeted the PD-1/PD-L1 pathway using anti-PD-1 or anti-PD-L1 antibodies have demonstrated the benefit of such treatments for patients with advanced UC, with these agents subsequently being approved by the Food and Drug Administration in the United States [16–18].

Better understanding of interactions between tumor cells and stromal cells in the tumor microenvironment may be important for assessing the immunologic characteristics of tumors. It was recently proposed that the tumor microenvironment can be classified into four categories on the basis of tumor cell PD-L1 expression and the presence or absence of TILs [19]. In addition, there have been several reports that TILs can be used to predict the prognosis of patients with UC, including UTUC [20–22]. Thus, it seems likely that tumor microenvironment immunity plays a pivotal role in determining the prognosis of UC. A few studies have examined both PD-L1 expression by tumor cells and the number of TILs in UC, but these factors have not been fully investigated in UTUC. Accordingly, we performed a retrospective study of both PD-L1 expression and TILs in UTUC, and we demonstrated that increased PD-L1 expression by tumor cells and a higher TIL density (TILD) were associated with progression of this cancer. These findings might shed new light on our understanding of the tumor microenvironment in UTUC.

## Materials and methods

### Patients

This retrospective study was performed in 79 patients (55 men and 24 women, median age: 71 years, range 42–85 years) with histopathologically diagnosed UC of the renal pelvis (*n* = 35) and ureter (*n* = 44) who underwent nephroureterectomy at Dokkyo Medical University Hospital between September 2004 and August 2015. All patients had preoperative CT and/or MRI for staging. Forty-one patients had lymph-node and/or distant metastases or were positive for lymphovascular invasion (LVI), and received postoperative adjuvant chemotherapy with GC or MVAC. The postoperative follow-up period ranged from 5 to 156 months, with a median of 45 months. For detection of metastatic disease, CT and/or MRI were performed every 2–4 months. Final assessment was done by review of the medical records in March 2019.

We collected data on the preoperative peripheral blood parameters, including the WBC, neutrophil, and lymphocyte counts, and the neutrophil-to-lymphocyte ratio (NLR).

### Assessment of TILs

**Fig. 1:**
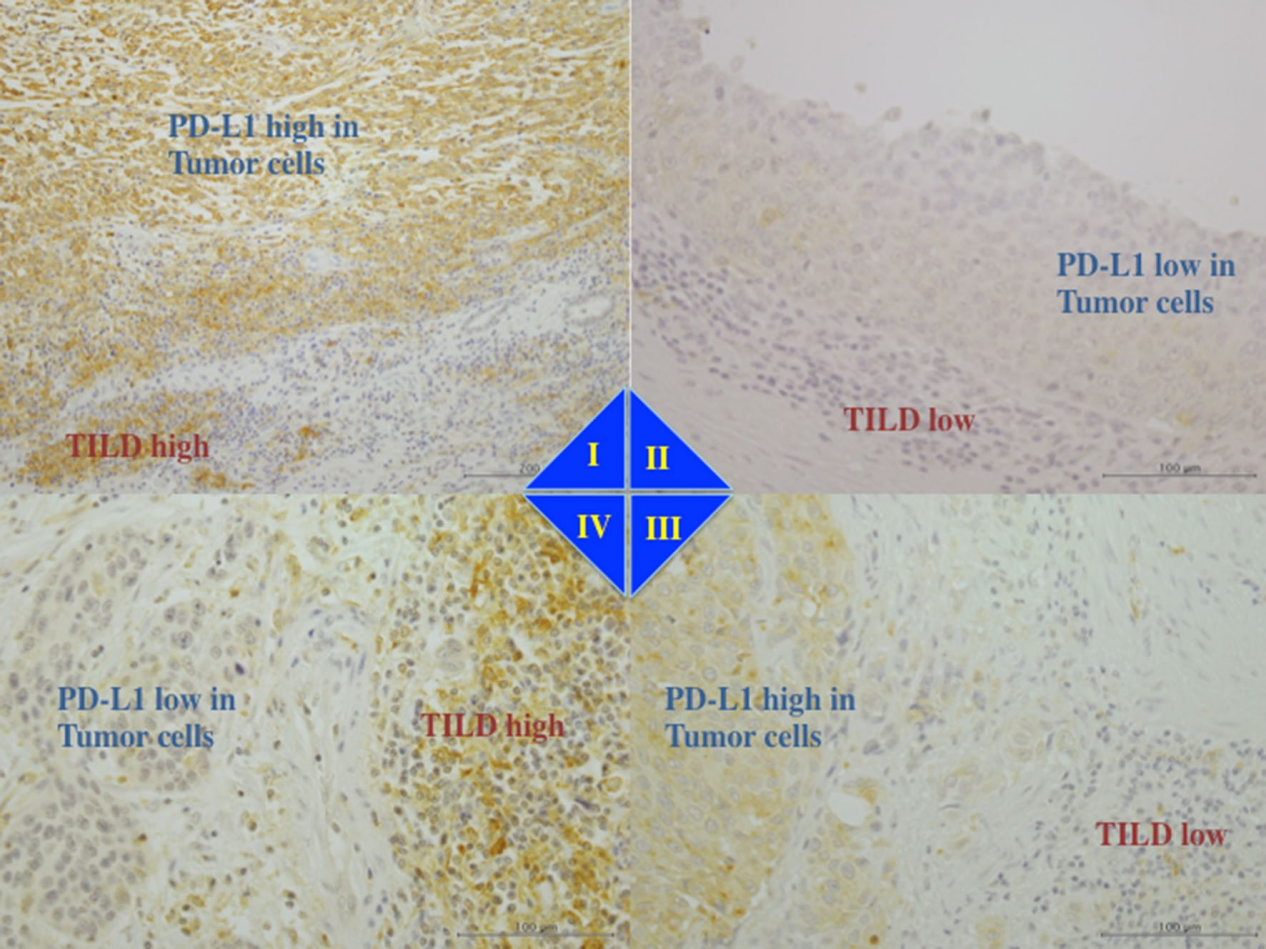
The level of PD-L1 expression in tumor cells as shown by immunohistochemistry and tumor-infiltrating lymphocyte density (TILD). The expression levels of PD-L1 and TILD in tumor cells were combined for classification into four groups i.e., type I (PD-L1 high and TILD high), type II (PD-L1 low and TILD low), type III (PD-L1 high and TILD low), and type IV (PD-L1 low and TILD high). PD-L1 expression level in TILs was not included in this classification. **I** Type I tumor showed high tumor cells PD-L1 expression and high TILD with high TILs PD-L1 expression. **II** Type II tumor showed low tumor cells PD-L1 and low TILD with low TILs PD-L1. **III** Type III tumor showed high tumor cells PD-L1 and low TILD with low TILs PD-L1. **IV** Type IV tumor showed low tumor cells PD-L1 and high TILD with high TILs PD-L1

In the present study, slides of full-face hematoxylin and eosin-stained sections from primary tumors were retrieved for the evaluation of TILs by light microscopy [22, 23]. Therein, we observed many mononuclear inflammatory cells in variable proportions, such as a small rounded cell and a large dark-stained nucleus with little eosinophilic cytoplasm. So far, TILs are evaluated by immunostaining with CD4 and CD8; however, the TILs are a mixture of pro-inflammatory immune cells including T cells, natural killer cells, dendritic cells (DCs), neutrophils, and macrophages [19]. Many mononuclear cells detected on the eosin-stained slides in this study were classified as TILs, using antibody against CD3 (PA0553, Leica Biosystems Newcastle Ltd, Newcastle, UK), CD4 (NCL-CD4-1F6, Leica Biosystems Newcastle Ltd, Newcastle, UK), CD8 (PA0183, Leica Biosystems Newcastle Ltd, Newcastle, UK), and CD25 (PA0305, Leica Biosystems Newcastle Ltd, Newcastle, UK) in formalin-fixed and paraffin-embedded tissues by immunohistochemical staining using the automated BOND system (Leica BOND-IIII system, Leica Biosystems Newcastle Ltd, Newcastle, UK) (Supplemental Figs. 1, 2). Thus, despite assessing the association of TILs with clinicopathological features by separating each immune cell type, the clinical usefulness of scoring TILs by combining these mononuclear cells into total TILs by hematoxylin and eosin staining has been demonstrated [24]. For standardizing the TILs scoring, we analyzed TILs on hematoxylin and eosin-stained slides prepared from the sections from the invasive front of primary tumor and areas surrounding the tumor, as per the guidelines developed by “International TILs Working Group 2014” [24]. Briefly, 5–7 slides, each having 1000 tumor cells and 1000 adjacent non-tumor cells, were examined in 5–10 microscopic fields, independently by two of the authors (Nukui and Kamai). The percentage of TILs was calculated from mononuclear cells within the invasive tumor cell nests, and the percentage of stromal TILs was calculated from mononuclear cells in stromal areas adjacent to the tumor (Fig. 1). As previously reported, different types of infiltrating immune cells can be located either in the tumor center, or at the invasive margin, or in the adjacent tertiary lymphoid structures, and the immune infiltrates can differ in different tumor types, or patients with the same cancer, or even within each patient [19]. Thus, no formal recommendation for clinically relevant TILs threshold was made by the “International TILs Working Group 2014” [24]. In this study, we set the cut-off value at 20%, and defined high TILD as either intratumoral or stromal TILs > 20%, as previously reported [25] (Supplemental Fig. 3).

### Immunohistochemistry

Immunohistochemical staining of specimens from the tumor tissue was performed to assess the PD-L1 expression by both, tumor cells and TILs. Formalin-fixed and paraffin-embedded tissue samples were cut into 4 μm-thick sections for immunohistochemistry using rabbit anti-PD-L1 antibody (EIL3N, Cell Signaling Technology, and Danvers, MA). Briefly, the sections were deparaffinized using xylene, followed by passage through graded ethanol series. Incubation with EDTA buffer was done for antigen unmasking, following which the sections were incubated in 3% hydrogen peroxide. After washing with Tris-buffered saline, sections were incubated overnight at 4 °C with the primary antibody. Subsequently, the expression of PD-L1 was detected using a ChemMate EnVision Detection Kit with DAB (Dako, Carpinteria, CA), according to the manufacturer’s instructions. All the 1000 tumor cells, from each patient, were counted in a high-power view by two of the authors independently (Nukui and Kamai). Furthermore, depending on the level of PD-L1 expression, we classified the tumors into two groups, namely a high expression group (> 5% positive tumor cells and > 5% positive TILs) and a low expression group (< 5% positive tumor cells and > 5% positive TILs) [23, 26].

Subsequently, we combined the tumor cell PD-L1 expression with TILD to classify tumors into four types as described earlier by Teng et al*. *[19], i.e., type I (high PD-L1 expression and high TILD), type II (low PD-L1 expression and low TILD), type III (high PD-L1 expression and low TILD), and type IV (low PD-L1 expression and high TILD). While this classification is a combination of tumor cell PD-L1 expression and TILD, the TILs PD-L1 expression has not been included.

### Statistical analysis

We used Fisher's exact test to investigate associations between two categorical variables for PD-L1/TILD status. The Mann–Whitney *U* test (two groups) or the Kruskal–Wallis test (three or more groups) was employed for assessment of the relationship between PD-L1/TILD status and preoperative peripheral blood parameters. Since the NLR cut-off points show heterogeneity in the literature [27], we divided NLR into two groups at the median value (2.436), the mean value (2.881), or the cut-off value obtained from time-dependent receiver-operating characteristic (ROC) curves (2.729) for assessment of survival. Curves for progression-free survival (PFS) and overall survival (OS) were drawn by the Kaplan–Meier method, and differences were assessed with the log-rank test. We examined prognostic factors with a potential influence on survival by using Cox regression analysis. Analyses were done with EZR software (Jichi Saitama Medical center, Saitama, Japan) [28], and *p* < 0.05 was taken to indicate statistical significance.

## Results

Based on the tumor cell PD-L1 expression and TILD, tumors were classified into four types (type I–IV). Representative examples of PD-L1 expression by both tumor cells and TILs are shown in Fig. 1. PD-L1 expression was positive in the cell membrane and/or cytoplasm of the tumor cells and TILs. Although TILs PD-L1 expression was not included in this classification [19], we found that within type I tumors (*n* = 17), nine tumors showed high TILs PD-L1 and eight tumors showed low TILs PD-L1; within type II tumor (*n* = 34), five tumors showed high TILs PD-L1 and 29 tumors showed low TILs PD-L1. Furthermore, within type III tumors (*n* = 14), two tumors showed high TILs PD-L1 and 12 tumors showed low TILs PD-L1, and within type IV tumors (*n* = 14), 11 tumors showed high TILs PD-L1 and 3 tumors showed low TILs PD-L1. We found a significant correlation between PD-L1 expression by tumor cells and TILD (*p* = 0.0334), as well as between PD-L1 expression by TILs and TILD (*p* < 0.0001). However, no such correlation was found between PD-L1 expression by both, tumor cells and TILs (*p* = 0.5723, Table 1).

**Table 1:** Interrelationship between PD-L1, TILD, and blood parameters/pathological characteristics

	Tumors		*p* value
	PD-L1 low	PD-L1 high	
TILD			
Low density	34	14	0.0334
High density	14	17	
TILs			
PD-L1 low	32	20	0.5723
PD-L1 high	16	11	

	TILD		*p* value
	Low density	High density	
TILs			
PD-L1 low	41	11	< 0.0001
PD-L1 high	7	20	

	Grade		T stage		LVI		NM stage	
	G1,2 (*n* = 32)	G3 (*n* = 47)	pTa,1 (*n* = 24)	pT2–4 (*n* = 55)	LVI (−) (*n* = 41)	LVI ( +) (*n* = 38)	N0M0 (*n* = 66)	N1–3 or M1 (*n* = 13)
Tumors								
PD-L1 low (*n* = 48)	25	23	21	27	30	18	42	6
PD-L1 high (*n* = 31)	7	24	3	28	11	20	24	7
* p* value	0.0107		0.0228		0.0012		0.5481	
TILs								
PD-L1 low (*n* = 52)	22	30	14	38	30	22	44	8
PD-L1 high (*n* = 27)	10	17	10	17	11	16	22	5
*p* value	0.8135		0.4415		0.1635		0.7533	
TILD								
Low density (*n* = 48)	27	21	19	29	32	16	43	5
High density (*n* = 31)	5	26	5	26	9	22	23	8
* p* value	0.0004		0.0438		0.0013		0.1183	

	WBC (mean ± S.D.)	Neutrophil (mean ± S.D.)	Lymphocyte (mean ± S.D.)	N/L ratio (mean ± S.D.)
Tumors				
PD-L1 low (*n* = 48)	6275 ± 2551	3956 ± 2287	1769 ± 731	2.607 ± 2.178
PD-L1 high (*n* = 31)	6964 ± 3102	4801 ± 2997	1552 ± 385	3.288 ± 2.294
*p* value	0.2743	0.0724	0.3372	0.0168
TILs				
PD-L1 low (*n* = 52)	6861 ± 3147	4546 ± 3072	1791 ± 618	2.808 ± 2.551
PD-L1 high (*n* = 27)	5937 ± 1790	2827 ± 1308	1478 ± 587	3.019 ± 1.507
*p* value	0.2137	0.4934	0.0491	0.1374
TILD				
Low density (*n* = 48)	6254 ± 1921	3874 ± 1435	1718 ± 641	2.402 ± 0.872
High density (*n* = 31)	6997 ± 3741	4961 ± 3726	1625 ± 598	3.635 ± 3.011
*p* value	0.6693	0.0851	0.5412	0.0217

PD-L1 expression by tumor cells demonstrated a significant positive correlation with a higher histological grade, higher pT stage, positive lymphovascular invasion (LVI), and a higher peripheral blood NLR (*p* = 0.0107, 0.0228, 0.0012, and 0.0168, respectively, Table 1). A higher TILD also showed a significant positive correlation with a higher histological grade, higher pT stage, positive LVI, and higher peripheral blood NLR (*p* = 0.0004, 0.0438, 0.0013, and 0.0217, respectively, Table 1). However, tumor cell PD-L1 expression and the TILD were not associated with lymph-node involvement or distant metastasis. There was also no relationship between PD-L1 expression by TILs and various clinicopathological factors. Compared with type I, type III, and type IV tumors, we found that type II tumors had a lower histological grade, a lower pT stage, were more likely to be negative for LVI, and had a lower peripheral blood NLR (*p* = 0.0002, 0.0068, 0.0002, and 0.0054, respectively, Table 2). On the other hand, a lower neutrophil count might be related to higher tumor PD-L1 expression and an increased TILD (*p* = 0.0724 and 0.0851, respectively, Table 1), and thus to type II tumors (*p* = 0.0763, Table 2). A lower peripheral lymphocyte count was related to higher PD-L1 expression in TILs (*p* = 0.0491, Table 1), while the WBC count was not related to the PD-L1/TILD status (Tables 1, 2).

**Table 2:** Relationship between PD-L1/TILD status and blood parameters/pathological characteristics

	Grade		T stage		LVI		NM stage	
	G1,2	G3	pTa,1	pT2–4	LVI (–)	LVI ( +)	N0M0	N1–3 or M1
	(*n* = 32)	(*n* = 47)	(*n* = 24)	(*n* = 55)	(*n* = 41)	(*n* = 38)	(*n* = 66)	(*n* = 13)
Type I: PD-L1 H/TILD H (*n* = 17)	2	15	0	17	5	12	13	4
Type II: PD-L1 L/TILD L (*n* = 34)	22	12	16	18	26	8	31	3
Type III: PD-L1 H/TILD L (n = 14)	5	9	3	11	6	8	12	2
Type IV: PD-L1 L/TILD H (*n* = 14)	3	11	5	9	4	10	10	4
*p* value	0.0008		0.0019		0.0014		0.2962	
Type II: PD-L1 L/TILD L (*n* = 34)	22	12	16	18	26	8	31	3
Other group (*n* = 45)	10	35	8	37	15	30	35	10
*p* value	0.0002		0.0068		0.0002		0.1361	

	WBC (mean ± S.D.)	Neutrophil (mean ± S.D.)	Lymphocyte (mean ± S.D.)	N/L ratio (mean ± S.D.)
Type I: PD-L1 H/TILD H (*n* = 17)	7176 ± 3806	4821 ± 3882	1528 ± 560	3.484 ± 2.990
Type II: PD-L1 L/TILD L (*n* = 34)	6067 ± 1860	3329 ± 1636	1663 ± 858	2.131 ± 0.661
Type III: PD-L1 H/TILD L (*n* = 14)	6707 ± 2061	4091 ± 1950	1360 ± 501	3.047 ± 0.996
Type IV: PD-L1 L/TILD H (*n* = 14)	6778 ± 3790	4067 ± 3883	1395 ± 940	3.837 ± 3.826
*p* value	0.5762	0.0874	0.0691	0.0826
Type II: PD-L1 L/TILD L (*n* = 34)	6067 ± 1860	3329 ± 1636	1663 ± 858	2.131 ± 0.661
Other group (*n* = 45)	6906 ± 3288	4359 ± 3345	1434 ± 677	3.448 ± 2.788
*p* value	0.1563	0.0763	0.2057	0.0054

**Fig. 2:**
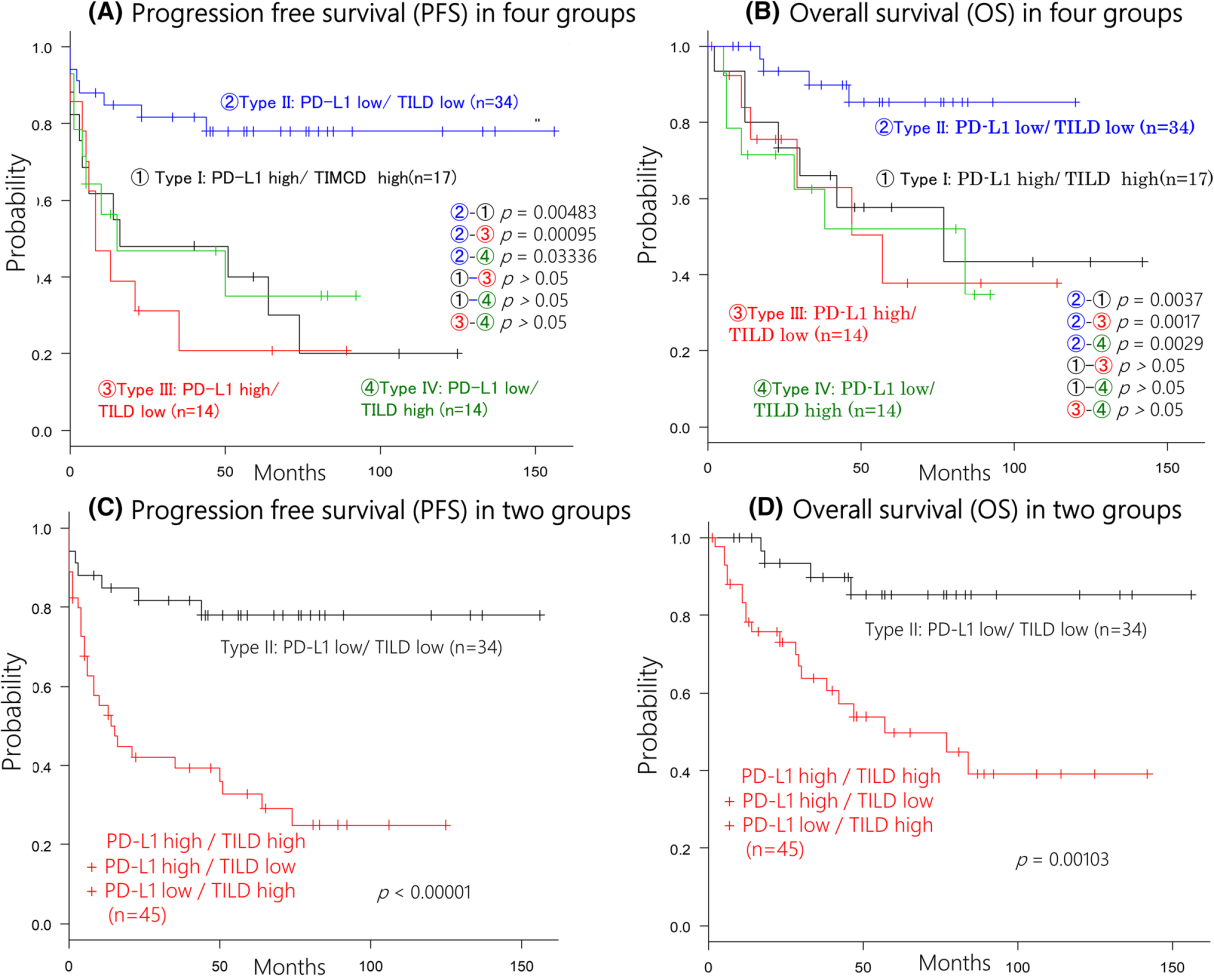
Survival curve in all patients. This survival curve is based on the status of PD-L1 in tumor cells and tumor-infiltrating lymphocytes density (TILD). **a**, **b** The tumors with type II (PD-L1 low and TILD low) showed a better progression-free survival (**a**) and overall survival than the other groups (**b**). **c**, **d** By combining the type I, III, and IV tumor into one group, the type II tumors were associated with a better progression-free survival (**c**) and overall survival (**d**)

Since patients with type II tumors showed longer survival (both PFS and OS) than patients with the other three types of tumors, while there were no differences of PFS and OS among the other three groups (Fig. 2a, b), we combined the latter three groups for comparison with the type II group. This analysis showed that the type II group had a significantly better PFS and OS than the combined group (*p* < 0.00001 and *p* = 0.00103, respectively, Fig. 2c, d). On the other hand, when we analyzed the survival by the three cut-off values of NLR (median, mean, and ROC), none of these cut-off values was associated with PFS or OS. Similarly, peripheral WBC, neutrophil, and lymphocyte counts had no influence on PFS or OS.

**Fig. 3:**
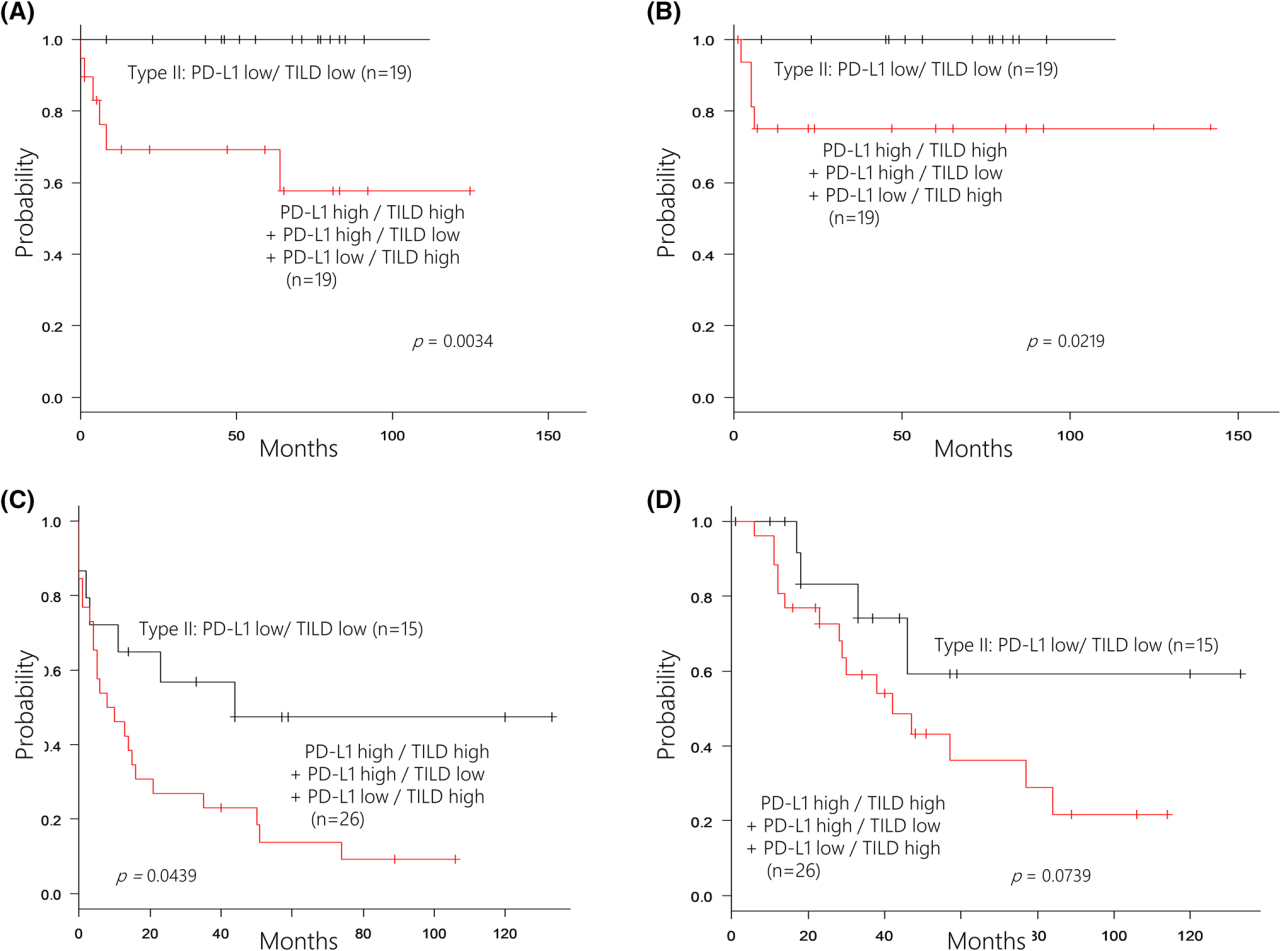
Relationship between the status of PD-L1 in tumor cells and tumor-infiltrating lymphocytes density (TILD) and effect of the adjuvant chemotherapy. In 38 patients who did not receive adjuvant chemotherapy, the patients with type II tumor had favorable progression-free survival (**a**) and overall survival (**b**). In 41 patients who received adjuvant chemotherapy, the patients with type II tumor had longer progression-free survival (**c**), but tendency toward a better overall survival (**d**)

Among 38 patients who did not receive adjuvant chemotherapy, those with type II tumors had significantly better PFS and OS than the other patients (*p* = 0.0034 and *p* = 0.0219, respectively, Fig. 3a, b). When we analyzed the influence of adjuvant chemotherapy on survival in the other 41 patients, PFS was significantly longer for those with type II tumors (*p* = 0.0439, Fig. 3c). OS was also better in type II patients, although the improvement was not significant (*p* = 0.0739) (Fig. 3d).

Univariate analysis identified the following factors as being associated with PFS: histological grade, LVI, pT stage, PD-L1 expression by tumor cells, TILD, and tumor type (based on PD-L1 expression by tumor cells and TILD). Multivariate analysis confirmed that the histological grade, LVI, and the tumor type were independent determinants of PFS (Table 3).

**Table 3:** Cox regression analysis for various potential prognostic factors in survival

Variable	Unfavorable/ favorable characteristics	No. of patients	Univariate analysis			Multivariate analysis		
			Relative risk	95% confidential interval	*p* value	Relative risk	95% confidential interval	*p* value
Progression-free survival								
Grade	3/2, 1	47/32	12.94	3.94–42.52	< 0.0001	10.16	2.53–40.85	0.0010
pT	4, 3, 2/1, a	55/24	6.44	1.96–20.89	0.0021	1.33	0.18–3.06	0.6848
LVI	1/0	38/41	3.83	1.87–7.84	0.0002	2.51	1.10–5.73	0.0295
PD-L1 in Tumor	High/low	31/48	2.91	1.49–5.66	0.0017	1.20	0.45–3.17	0.7152
TILD	High/low	31/48	2.14	1.12–4.13	0.0231	2.19	0.88–5.46	0.0914
PD-L1 in TILs	High/low	27/52	1.19	0.44–1.83	0.7672	2.17	0.20–4.03	0.0595
Combination of PD-L1 in tumor and TILD	Type I, III, IV/type II	45/34	4.54	1.98–10.42	0.0004	4.83	1.14–20.36	0.0318
Overall survival								
Grade	3/2, 1	47/32	10.96	2.56–46.97	0.0013	15.43	2.03–115.40	0.0081
pT	4, 3, 2/1, a	55/24	3.52	1.04–11.74	0.0430	4.55	0.43–4.33	0.0860
LVI	1/0	38/41	3.11	132—7.32	0.0094	1.81	0.65—5.07	0.2588
PD-L1 in Tumor	High/low	31/48	2.29	1.03–5.12	0.0434	1.72	0.54–5.48	0.3615
TILD	High/low	31/48	2.51	1.11–5.65	0.0267	1.30	0.39–4.39	0.6720
PD-L1 in TILs	High/low	27/52	1.05	0.45–2.46	0.9047	2.33	0.16–1.10	0.0774
Combination of PD-L1 in tumor and TILD	Type I, III, IV/type II	45/34	5.04	1.72–14.76	0.0032	6.23	1.04–37.44	0.0456

According to univariate Cox analysis of factors associated with OS, the histological grade, pT stage, PD-L1 expression by tumor cells, TILD, and tumor type (based on PD-L1 expression by tumor cells and TILD) were all prognostic indicators, but only the histological grade and tumor type were significant determinants according to multivariate analysis (Table 3).

## Discussion

Increased PD-L1 expression by tumor cells is essential for development and maintenance of immune tolerance and facilitates evasion of the host immune system, suggesting that PD-L1 blockade is a potential anticancer strategy [12–14]. Anti-PD1/PD-L1 therapy has recently been developed as immunotherapy for several cancers, including advanced UC. The antitumor effect of atezolizumab (an anti-PD-L1 antibody) was reported to depend on the level of PD-L1 expression by tumor-infiltrating immune cells [16]. On the other hand, the antitumor effect of pembrolizumab (an anti-PD-1 antibody) is not related to PD-L1 expression by the tumor and/or infiltrating immune cells [17], and the antitumor activity of nivolumab (another anti-PD-1 antibody) is not influenced by tumor cell PD-L1 expression [18]]. In addition, it has been reported that UC of the bladder (BUC) patients with high PD-L1 expression tends to have advanced cancer and a poor prognosis [29–31], but there have been conflicting reports that high PD-L1 expression is associated with a better prognosis [32]. Thus, the prognostic influence of tumor PD-L1 expression remains controversial. However, it seems that immunotherapy should simultaneously target both the tumor itself and the stromal components for maximum effect, suggesting the importance of assessing both tumor cells and TILs in the tumor microenvironment.

Interactions between tumor cells and the immune system are complex. It is generally accepted that understanding interactions between host immunity and the tumor microenvironment may be important for unraveling the biology of cancer immunology. All types of immune cells may potentially be found in the tumor microenvironment, such as tumor-infiltrating immune cells (TIICs) that include macrophages, neutrophils, dendritic cells, mast cells, natural killer cells, naive and memory B cells, and effector T cells (T helper cells, regulatory T cells, and cytotoxic T cells). Also, the region infiltrated by these TIICs is quite wide, and can include the central tumor zone, invasive margin, and/or peripheral tumor stroma. Furthermore, the effects of these cells depend on their type, density, and location, as well as their function (which is influenced by regulatory chemokines and cytokines) [33]. Therefore, the current evidence suggests that TILs represent the interaction between the immune system and tumor microenvironment, with both PD-L1 expression and the TILD influencing the survival of cancer patients [34, 35].

Upregulation of PD-L1 expression by two immune resistance mechanisms may be involved in evasion of host immunity by solid cancer and/or development of immune tolerance. PD-L1 plays a dominant role in suppressing in vivo effector T-cell responses, especially in the tumor microenvironment, and PD-L1 expression in this microenvironment is thought to be regulated by tumor-associated stroma (adaptive immune resistance) and/or tumor cells (intrinsic immune resistance) [14, 36]. Recently, Teng et al*. *[19] proposed that cancers can be categorized into four types for tailoring immunotherapy based on tumor cell PD-L1 expression and the presence or absence of TILs in the tumor microenvironment. These are type I cancer (PD-L1( +) tumor cells and TIL( +); adaptive immune resistance), type II cancer (PD-L1(−) tumor cells and TIL(−); immunologic ignorance), type III cancer (PD-L1( +) tumor cells and TIL(−); intrinsic induction), and type IV cancer (PD-L1(−) tumor cells and TIL( +); tolerance). This classification provides a platform for discussing the optimum immunotherapy strategy for targeting the four different tumor types based on immune checkpoint blockade. For example, approximately 38% of patients with advanced melanoma have type I tumors and may be more likely to respond to immune checkpoint blockade [19, 37]. Their tumors will most probably benefit from single-agent anti–PD-1/L1 therapy, because they contain intratumoral T cells that have been inactivated by PD-L1 engagement. On the other hand, another large group of melanoma patients (41%) have type II tumors and their prognosis is predicted to be very poor based on the lack of a detectable immune reaction. According to Teng et al. [19], these type II patients with an immune ignorant phenotype will have a poor outcome regardless of their treatment. In contrast, we observed that UC patients with type II tumors (low tumor cell PD-L1 expression and low TILD) had a lower histological grade and lower pT stage than the other three groups, as well showing a favorable prognosis. Similarly, it has been reported in BUC that lower PD-L1 expression by tumor cells and a lower TILD are related to higher histological differentiation, noninvasiveness, and better survival [23]. Thus, our findings in UTUC may be in the same line than those in BUC in general. Type II tumors might be considered to show innate immune resistance, which means that constitutive activation of oncogenic signaling up-regulates PD-L1 expression by tumor cells in the tumor microenvironment independently of inflammatory signals [14, 38], suggesting that the pathways up-regulating PD-L1 are not so highly activated in type II UTUC. It has been reported that tumors with higher PD-L1 expression may have a greater mutational burden (and mutation-associated neoantigen-specific T cells). In recent genome-wide expression and sequencing studies, comprehensive molecular characterization has indicated that BUC expresses more somatic mutations and high levels of tumor-specific neoantigens [39, 40], while UTUC shows a differing frequency of mutations from BUC [41]. On the other hand, type I tumors are affected by adaptive resistance, which suggests that inflammatory signals such as interferons secreted by CD4^+^ T helper cells and CD8^+^ T cells in the tumor microenvironment induce upregulation of PD-L1 expression by tumor cells and TIICs, including macrophages, myeloid suppressor cells, dendritic cells, and even lymphocytes. These changes could trigger immunosuppression as a result of crosstalk between tumor cells and TIICs in the tumor microenvironment, because PD-L1 suppresses T-cell migration/proliferation and also prevents cancer cell killing by binding to T-cell receptors [14]. In the present study, we investigated the level of PD-L1 expression by TILs. We initially hypothesized that PL-L1 expression by TILs might be associated with progression of UTUC. However, we found that PL-L1 expression by TILs was positively associated with the TILD, but was not correlated with the histological grade, pT stage, LVI, or prognosis of UTUC. Wang et al. recently reported that higher PD-L1 expression by both tumor cells and TILs was related to worse survival in patients with BUC, while higher PD-L1 expression by TILs was an independent indicator of a shorter prognosis, suggesting that the level of PD-L1 expression by TILs influenced tumor progression [23]. Taken together, these findings suggest that tumor-specific and/or organ-specific changes of TILs and regulation of PD-L1 expression may occur. Therefore, we should study the mutational burden of UTUC and assess its relation with PD-L1 expression and the TILD, as well as evaluating the response to PD-1/PD-L1 blockade.

In addition to lymphocytes, other key TIICs include macrophages, myeloid-derived suppressor cells (MDSCs), and DCs. Macrophages are key components of the tumor microenvironment and PD-L1 is usually expressed by a subset of macrophages [36, 42]. A recent study revealed upregulation of PD-L1 expression by human and murine macrophages and DCs in both the tumor microenvironment and the draining lymph nodes, with PD-L1 expression by macrophages and DCs in the tumors of melanoma and ovarian cancer patients being associated with the response to immunotherapy using anti-PD-1 antibody alone or the combination of anti–PD-1 and anti-cytotoxic T-lymphocyte-associated antigen-4 (CTLA-4) antibodies [43]. Tumor-associated macrophages (TAMs) represent the major inflammatory cell population in tumors and orchestrate various aspects of the response to cancer [44]. During the development of adaptive immune resistance, macrophages are activated by interferons and kill tumor cells, but the same macrophages may also protect the tumor against attack from infiltrating T cells by expressing inhibitory PD-L1 [42]. Since malignant cells and TAM show considerable cytological pleiomorphism, accurately identifying individual cell types based on morphology alone might be challenging. Accordingly, we should perform immunohistochemical analysis of immune cells infiltrating tumor tissues and the interplay among PD-L1, Akt, and interferons in the tumor microenvironment of patients receiving immunotherapy that targets the PD-1/PD-L1 pathway to further elucidate the interactions between immune cells and tumor cells.

Tumors express a wide range of antigens, including self-antigens, and are infiltrated by (CD4^+^CD25^+^Foxp3^+^) regulatory T cells (Tregs) and MDSCs that actively inhibit T-cell responses through direct cell–cell contact [45]. Tregs play an essential role in promoting immunosuppression and self-tolerance of tumor antigens in cancer patients, as well as in the development of tolerance to microbial antigens in patients with chronic infection [45]. MDSCs with the phenotypic features of partially differentiated granulocyte macrophages and monocytic lineage myeloid precursors show a marked increase in the peripheral blood of tumor-bearing animals and cancer patients. Under certain experimental conditions, these progenitors undergo differentiation into antigen-presenting cells, such as macrophages and DCs [46].

There is a growing body of evidence to suggest that the systemic inflammatory response (SIR) in the tumor microenvironment is closely related to unfavorable outcomes in cancer patients. Among several SIR-related hematological factors, the NLR is an inflammatory index that has been considered as a potential prognostic factor in human cancer, and a high peripheral blood NLR is associated with poor overall survival in many solid tumors, including UTUC [27, 47, 48]. Although the mechanisms underlying the association of a high NLR with a poor outcome of cancer are not fully understood, one possibility is cytokine-mediated local immunosuppression, in which neutrophils recruited and activated by an inflammatory response inhibit the immune system by suppressing the cytolytic activity of immune cells such as lymphocytes, activated T cells, and natural killer cells [27, 49]. In the present study, when we divided the patients into two groups by the NLR (median value, mean value, or cut-off value obtained from ROC curves), no differences of survival time were observed among any of these pairs of groups. However, a lower tumor cell PD-L1 expression and a lower TILD were associated with a lower NLR. Patients with type II tumors had a lower NLR and showed a better survival compared to the other groups, indicating that NLR may be related to PD-L1 expression and TILD in the tumor microenvironment in UTUC. Although examining the PD-L1/TILD status may be needed for more detailed assessment of the tumor microenvironment, we could not repeatedly obtain tumor tissue specimens. Since the peripheral blood NLR can be measured conveniently, it may be more useful for predicting the response to anticancer therapy and as a prognostic indicator. In the literature published to date, heterogeneity of the NLR cut-off points is evident [27]. To determine the optimal cut-off point for clinical use in guiding treatment decisions, further investigation will be necessary to more precisely evaluate how NLR reflects the PD-L1/TILD status in the tumor microenvironment.

The present study had several limitations, including its retrospective design, a relatively small number of subjects, and a follow-up period that was too short to allow definite conclusions regarding the possible influence of PD-L1 expression and the TILD on progression of UTUC. Therefore, our findings need to be confirmed by a large-scale prospective controlled clinical trial. It is generally thought that the effects of immunotherapy depend on the following three factors: (1) elevated expression of PD-L1 by tumor cells; (2) a higher tumor mutational burden associated with targeting by mutation-associated neoantigen-specific T cells, and (3) greater tumor infiltration by TILs. Therefore, to elucidate the interactions between immune cells and tumor cells, further investigation of oncogenic signaling pathways, the tumor mutational burden, and mutation-associated neoantigen-specific T cells seems to be warranted in patients with UTUC, including the types of immune cells infiltrating tumor tissues and interrelations among PD-L1, TAMs, MDSCs, and Tregs in the tumor microenvironment. It is not only important to understand the immunologic characteristics of the tumor microenvironment, but also to investigate how data on PD-L1 expression/TILD in surgical specimens can be utilized to develop new therapeutic strategies for cancer.

### Electronic supplementary material

Below is the link to the electronic supplementary material.

Supplemental figure 1: Tumor-infiltrating lymphocytes (TILs) in grade 3 and invasive non-papillary tumor. *HE* Hematoxylin and eosin-stained slide. Representative images of immunohistochemical detection of CD4, CD8, and CD25 (brown) in TILs (PDF 913 kb)

Supplemental figure 2: Tumor-infiltrating lymphocytes (TILs) in grade 1/2 and non-invasive papillary tumor. *HE* Hematoxylin and eosin-stained slide. Representative images of immunohistochemical detection of CD4 and CD8 (brown) in TILs. CD25 positive TILs are very little (PDF 862 kb)

Supplemental figure 3: Assessment of tumor-infiltrating lymphocyte density (TILD). Hematoxylin and eosin-stained slide. TILs infiltration is extremely sparse (**a**) and weakly (**b**) in lower histological grade and non-invasive papillary tumors, showing low TILD. TILs infiltrate extensively in high grade and invasive non-papillary tumors (**c**, **d**), displaying high TILD (PDF 1003 kb)

## Abbreviations

BUC: Urothelial carcinoma of the bladder
CD: Cluster of differentiation
CT: Computed tomography
CTLA-4: Cytotoxic T-lymphocyte-associated antigen-4
DCs: Dendritic cells
GC: Gemcitabine and cisplatin
LVI: Lymphovascular invasion
MDSC: Myeloid-derived suppressor cell
MVAC: Methotrexate, vinblastine, doxorubicin, and cisplatin
NLR: Neutrophil-to-lymphocyte ratio
OS: Overall survival
PD-1: Programmed cell death protein 1
PD-L1: Programmed cell death 1 ligand 1
PFS: Progression-free survival
pT: Pathological tumor
ROC: Receiver-operating characteristic
S.D.: Standard deviation
SIR: Systemic inflammatory response
TAM: Tumor-associated macrophage
TIIC: Tumor-infiltrating immune cell
TIL: Tumor-infiltrating lymphocyte
TILD: Tumor-infiltrating lymphocyte density
TNM: Tumor lymph-node metastasis classification of malignant Tumors
Tregs: Regulatory T cells
UC: Urothelial carcinoma
UTUC: Upper tract urothelial carcinoma
