# Supplementary figures and images for: Association of cancer progression with elevated expression of programmed cell death protein 1 ligand 1 by upper tract urothelial carcinoma and increased tumor-infiltrating lymphocyte density
Source: Cancer Immunol Immunother. 2020 Feb 6;69(5):689–702. doi: 10.1007/s00262-020-02499-7 (PMC7183489; doi:10.1007/s00262-020-02499-7)

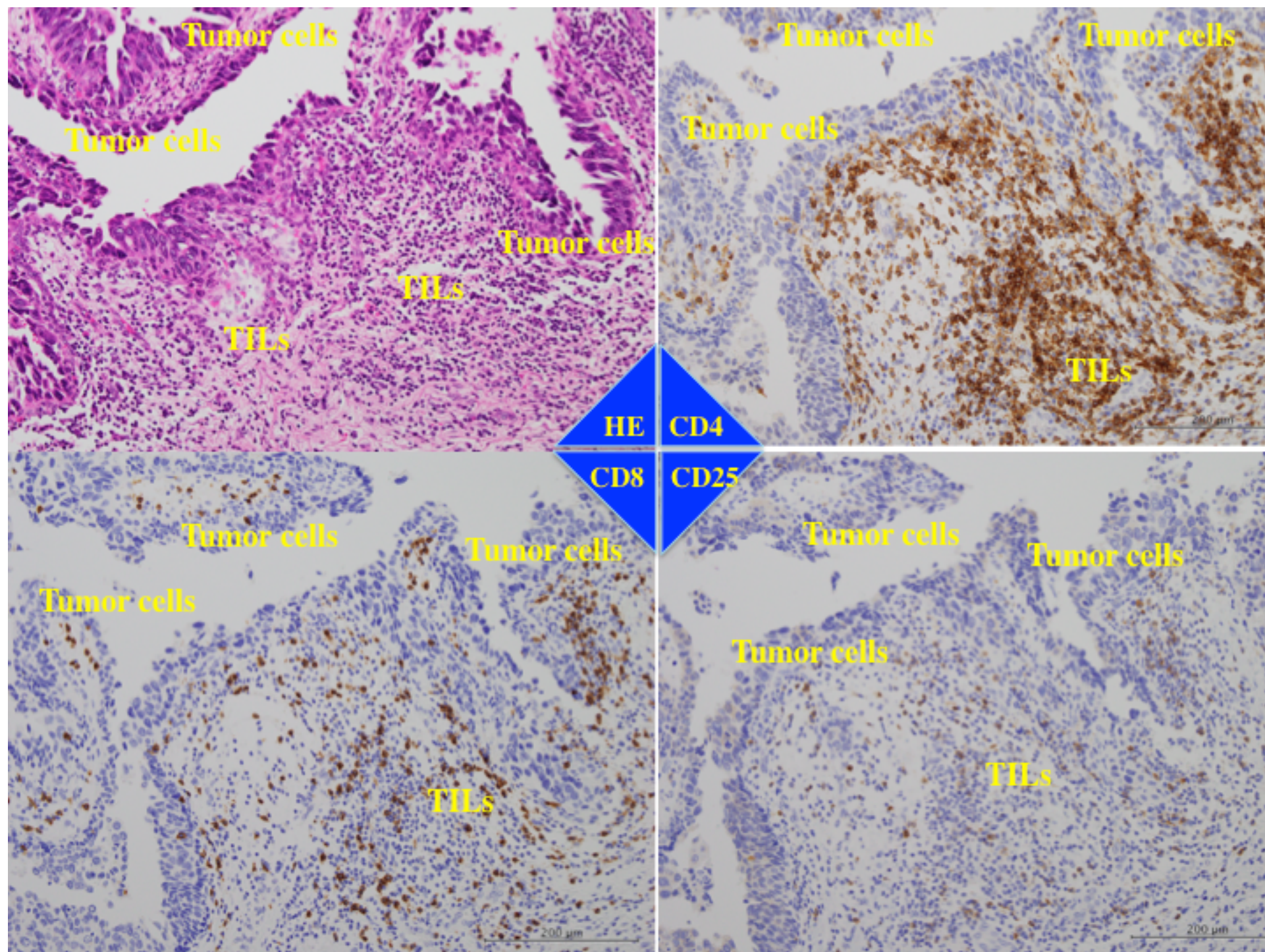

Supplement: Supplementary file 1 — Supplemental figure 1: Tumor-infiltrating lymphocytes (TILs) in grade 3 and invasive non-papillary tumor. HE Hematoxylin and eosin-stained slide. Representative images of immunohistochemical detection of CD4, CD8, and CD25 (brown) in TILs (PDF 913 kb) [file 262_2020_2499_MOESM1_ESM.pdf]

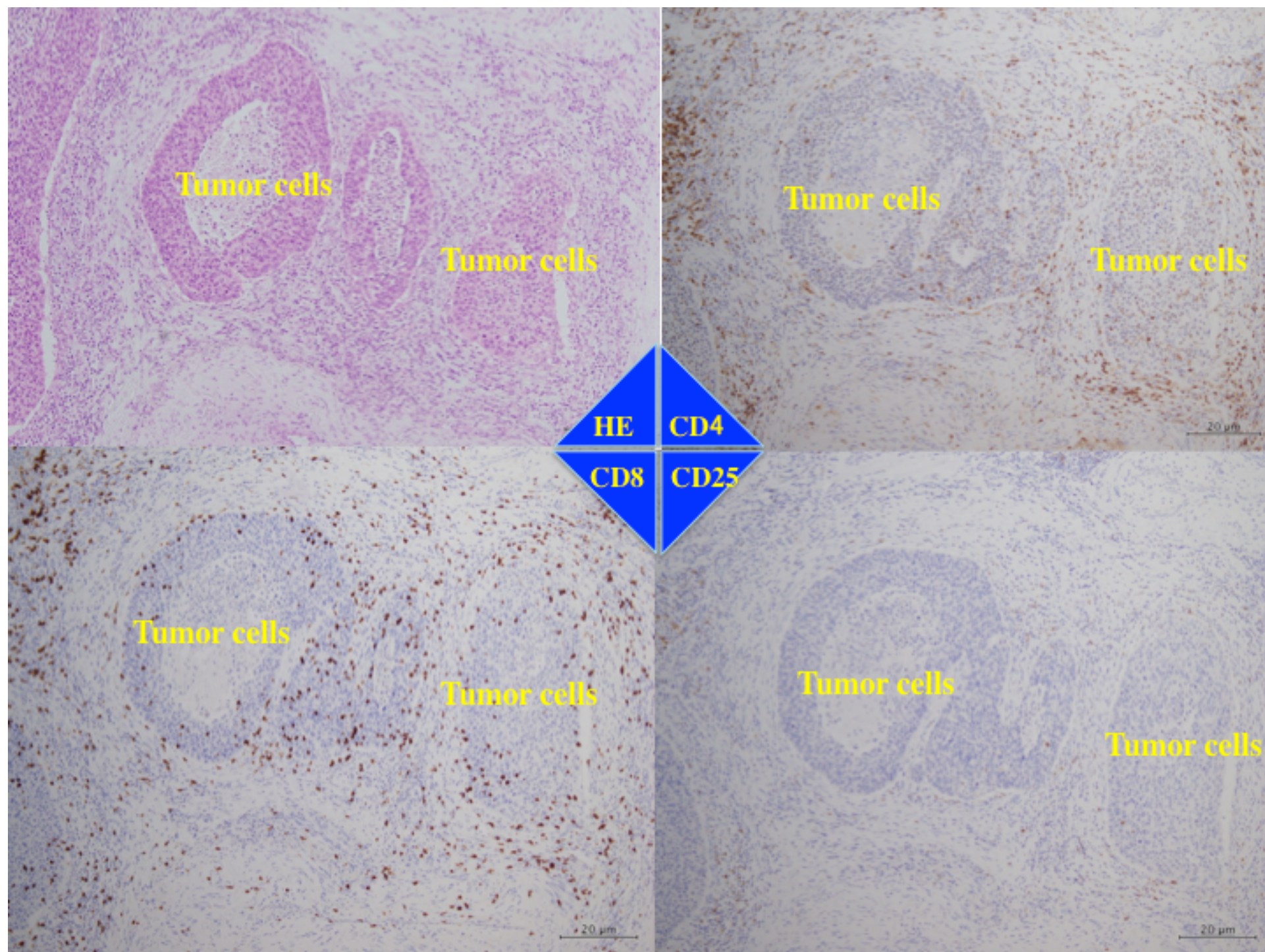

Supplement: Supplementary file 2 — Supplemental figure 2: Tumor-infiltrating lymphocytes (TILs) in grade 1/2 and non-invasive papillary tumor. HE Hematoxylin and eosin-stained slide. Representative images of immunohistochemical detection of CD4 and CD8 (brown) in TILs. CD25 positive TILs are very little (PDF 862 kb) [file 262_2020_2499_MOESM2_ESM.pdf]

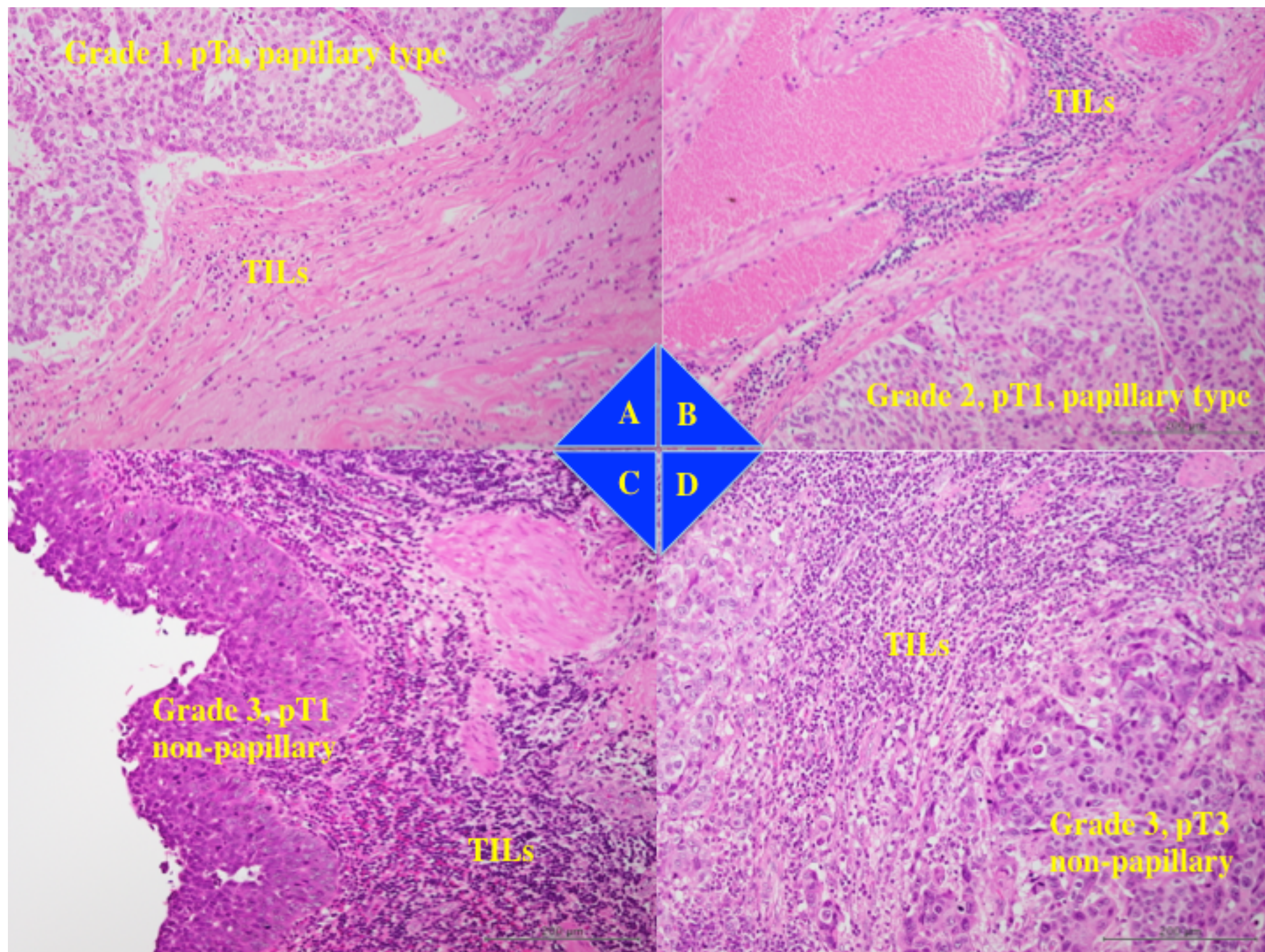

Supplement: Supplementary file 3 — Supplemental figure 3: Assessment of tumor-infiltrating lymphocyte density (TILD). Hematoxylin and eosin-stained slide. TILs infiltration is extremely sparse (a) and weakly (b) in lower histological grade and non-invasive papillary tumors, showing low TILD. TILs infiltrate extensively in high grade and invasive non-papillary tumors (c, d), displaying high TILD (PDF 1003 kb) [file 262_2020_2499_MOESM3_ESM.pdf]
